# 50 Racial and Ethnic Disparities in Complications Following Burn Injury in Adult Patients

**DOI:** 10.1093/jbcr/irae036.074

**Published:** 2024-04-17

**Authors:** Mecklin V Ragan, Christian Mpody, Samantha J Wala, Kelli Patterson, Olubukola Nafiu, Rajan K Thakkar, Dana M Schwartz

**Affiliations:** Nationwide Children’s Hospital, Falls Church, Virginia; Nationwide Childrens Hospital, Powell, Ohio; Nationwide Children’s Hospital, Columbus, OH; Nationwide Children’s Hospital, Falls Church, Virginia; Nationwide Childrens Hospital, Powell, Ohio; Nationwide Children’s Hospital, Columbus, OH; Nationwide Children’s Hospital, Falls Church, Virginia; Nationwide Childrens Hospital, Powell, Ohio; Nationwide Children’s Hospital, Columbus, OH; Nationwide Children’s Hospital, Falls Church, Virginia; Nationwide Childrens Hospital, Powell, Ohio; Nationwide Children’s Hospital, Columbus, OH; Nationwide Children’s Hospital, Falls Church, Virginia; Nationwide Childrens Hospital, Powell, Ohio; Nationwide Children’s Hospital, Columbus, OH; Nationwide Children’s Hospital, Falls Church, Virginia; Nationwide Childrens Hospital, Powell, Ohio; Nationwide Children’s Hospital, Columbus, OH; Nationwide Children’s Hospital, Falls Church, Virginia; Nationwide Childrens Hospital, Powell, Ohio; Nationwide Children’s Hospital, Columbus, OH

## Abstract

**Introduction:**

Burn injury continues to contribute to significant and preventable morbidity and mortality in the United States. Despite an increased focus on racial and ethnic disparities in healthcare, there remains a critical knowledge gap in our understanding of the effect of these disparities on complications in the adult burn population.

**Methods:**

The American Burn Association’s National Burn Repository data were reviewed from 2010-2018. Information regarding demographics, burn mechanism and severity, complications, and clinical outcomes were recorded. Data analysis was performed using 1:1 propensity-score-matching and logistic regression modeling.

**Results:**

Among 215,071 adult patients with burn injury, racial distribution was as follows: 65.2% White, 19.1% Black, 2.2% Asian, 0.7% American Indian/Alaskan Native, and 12.8% Other (including Native Hawaiian/Other Pacific Islander, and multiple races). Flame injuries were the most common cause of burns across all races (35.2%), followed by scald burns (23.3%). Most patients, regardless of race, had partial thickness burns. Black and Asian patients were more likely to die compared to White patients (OR 1.10, CI 1.00-1.21; OR 1.35, CI 1.03-1.76, respectively). Compared to their White peers, Black patients were more likely to develop in-hospital complications (OR 1.12, CI 1.06-1.17), while complication rates were lower for American Indian/Alaskan Native and those classified as “Other” (OR 0.93, CI 0.87-0.99; OR 0.080 CI 0.66-0.97, respectively). Black patients, when compared to White patients, were more likely to sustain cardiovascular (OR 1.27 CI 1.13-1.43), gastrointestinal (OR 1.24 CI 1.13-1.43), infectious (OR 1.63 CI 1.45-1.834), and electrolyte/other complications (OR 1.10, CI 1.02-1.20). Patients categorized as Other were less likely to have cardiovascular (OR 0.82 CI 0.69-0.97) and electrolyte/other complications compared to White patients (OR 0.97, CI 0.79-1.00). Black, Asian, and patients categorized as Other had significantly increased length of hospital stay (OR 1.17, CI 1.14-1.21; OR 1.21, CI 1.14-1.29; OR 1.21, CI 1.17-1.25, respectively) and longer duration of mechanical ventilation (OR 1.36, CI 1.26-1.47; OR 1.28, CI 1.04-1.58; OR 1.13, CI 1.02-1.25, respectively). All minority groups had a significantly increased length of ICU stay (OR 1.36, CI 1.26-1.47; OR 1.28, CI 1.04-1.58; OR 1.13, CI 1.02-1.25, respectively) compared to White patients.

**Conclusions:**

We showed disparities in the post-injury complication rates among adult burn patients. These findings highlight the need for further work to determine the etiology of these disparities to improve burn care for all patients, and to develop systems to prevent their persistence.

**Applicability of Research to Practice:**

We demonstrated disparities in adult burn patient care impacts complication rates in this population, highlighting the need for further research and system improvements to prevent the persistence of these disparities.

